# The Influence of Socioeconomic Status on Children’s Brain Structure

**DOI:** 10.1371/journal.pone.0042486

**Published:** 2012-08-03

**Authors:** Katarzyna Jednoróg, Irene Altarelli, Karla Monzalvo, Joel Fluss, Jessica Dubois, Catherine Billard, Ghislaine Dehaene-Lambertz, Franck Ramus

**Affiliations:** 1 Département d’Etudes Cognitives, Ecole Normale Supérieure, Paris, France; 2 Nencki Institute of Experimental Biology, Warsaw, Poland; 3 Cognitive Neuroimaging Unit, Institut National de la Santé et de la Recherche Médicale, Gif sur Yvette, France; 4 Neurospin, Comissariat à l’Energie Atomique, Division of Life Sciences, Institute of BioImaging, Gif sur Yvette, France; 5 University Paris 11, Orsay, France; 6 Assistance Publique-Hôpitaux de Paris, Le Kremlin-Bicêtre, Paris, France; 7 Neurologie pédiatrique, Hôpitaux Universitaires Genève, Genève, Suisse; Université de Montréal, Canada

## Abstract

Children’s cognitive abilities and school achievements are deeply affected by parental socioeconomic status (SES). Numerous studies have reported lower cognitive performance in relation to unfavorable environments, but little is known about the effects of SES on the child’s neural structures. Here, we systematically explore the association between SES and brain anatomy through MRI in a group of 23 healthy 10-year-old children with a wide range of parental SES. We confirm behaviorally that language is one of the cognitive domains most affected by SES. Furthermore, we observe widespread modifications in children’s brain structure. A lower SES is associated with smaller volumes of gray matter in bilateral hippocampi, middle temporal gyri, left fusiform and right inferior occipito-temporal gyri, according to both volume- and surface-based morphometry. Moreover, we identify local gyrification effects in anterior frontal regions, supportive of a potential developmental lag in lower SES children. In contrast, we found no significant association between SES and white matter architecture. These findings point to the potential neural mediators of the link between unfavourable environmental conditions and cognitive skills.

## Introduction

Socioeconomic status (SES) is a multidimensional construct that includes not only measures of material wealth, but also education and social prestige. Parental SES can affect an individual from very early development in utero as well as throughout life. Stress, nutrition, parental care and cognitive stimulation have been suggested as some of the factors that mediate the impact of SES on both brain structures and cognitive functions across development [1], [2]. At least three cognitive systems (i.e. language, executive function and memory) have been suggested to be influenced by SES [3], [4]. Language abilities – including vocabulary, literacy, phonological awareness and syntax – are strongly correlated with SES [5], [6], [7], [8]. Low-SES children also perform more poorly than their peers from high/middle SES on tasks probing selective attention, inhibition, cognitive control and working memory [9], [10], [11], [12].

Although a substantial body of work has focused on elucidating the cognitive impact of a child’s living environment, there is much more limited understanding of the neural mediators of these effects. The goal of the present study is to provide further evidence for these neural mediators. So far only three studies have investigated structural brain differences associated with SES. Hanson and colleagues [13] explored the relation between household income and hippocampi and amygdalae using a region of interest (ROI) approach in voxel based morphometry (VBM), in a large scale (n = 317) study of children (4–18 year olds). They showed that children from families with lower income had less gray matter in bilateral hippocampi than children from families with higher income. This relation was not significant for amygdalae, consistently with the authors’ hypothesis of a specific involvement of the hippocampi in stress regulation and long-term memory. Another study [14] looked at the association between subjective social status in adults and gray matter volume in three regions of interest: hippocampi, amygdalae and anterior cingulate cortex. Individual differences in subjective social status, but not conventionally defined SES, were associated with gray matter volume in the anterior cingulate cortex. Those subjects who viewed themselves higher on social ladder had more gray matter volume in this area. No such association was found in hippocampi or amygdalae.

Finally a recent study [15] aimed at determining the effects of genetic and of environmental factors such as age, sex, SES and IQ on white matter microstructure measured by fractional anisotropy (FA). In adults, a socioeconomic index based only on occupation was not significantly associated with FA, however it interacted with genes that affect white matter integrity.

Thus, the few studies that have up to now investigated the neural mediators of the effects of SES have all focused on specific regions of interest. They have entirely overlooked brain regions that would be prime candidates for certain effects of SES – on language for instance. In the present study, we aimed to further elucidate these neural mediators without any a priori focus on specific brain regions. We undertook exploratory whole-brain analyses on 23 healthy children within a narrow age range and showing a large variability in parental SES. We analysed gray matter properties using both voxel-based morphometry (VBM) and surface-based morphometry (SBM) based on T1-weighted MRI sequences, as well as white matter microstructure using tract based spatial statistics (TBSS). The existing literature suggests a few plausible predictions. Firstly, differences in the hippocampus and parts of the prefrontal cortex are particularly expected, given their association with the stress response system in humans [16], [17] and other animals [18], [19], [20] and according to the previous ROI-based study [13]. Secondly, since language has been shown to be one of the cognitive systems most highly influenced by SES, we may expect effects in language-related (left perisylvian) regions. Similarly, given the reported behavioral effects of SES on memory and executive functions, we may also anticipate structural differences in the hippocampus, in medial temporal lobes and in the prefrontal cortex [3]. Considering previous evidence [15], we are not predicting an association between white matter microstructure and SES, although the effects on children and adults might differ.

## Materials and Methods

### Participants

Participants were recruited from various schools in the Paris area and selected so as to maximize the range of socioeconomic status. Twenty-three healthy children (13 girls, 10 boys; age range = 8 years 11 months −10 years 10 months; mean age = 9 years 7 months, SD = 6 months) with no identified learning disability and having normal education were submitted to an MRI exam, comprising structural and functional sequences. A comparison of these children with dyslexic children is reported in another study by Monzalvo et al. [21]. All children and their parents gave written informed consent prior to being tested and the experimental procedures were approved by the ethical committee from Bicêtre Hospital. Children were included in the study if they were born at term (gestational age >36 weeks). Information about birth weight was available for a subset of 19 children (mean = 3167 g; SD = 747.4 g, range 1500–4230 g). Medical history and health status was collected. Children who suffered from chronic illness of any kind, including psychiatric and neurological disorders, were excluded from the study.

SES data were acquired from mothers’ responses to a sociodemographic questionnaire. Maternal education and current profession were used as indicators of SES, since mothers were identified as the only caregivers in a number of families (6 out of 23), and consistently with previous studies [12]. The level of education and occupational status were coded from 1 to 7 and 1 to 8 respectively (highest to lowest), in accordance with the manual of the Hollingshead Two-Factor Index of Social Position [22] (see Table 1). Direct information about income was not allowed to be collected by the ethical committee. SES scores were derived by summing the occupation status, which had a weight of 7, and the education status, which had a weight of 4. The corresponding SES index ranged from 84 (when the mother was unemployed and had no formal education beyond obligatory schooling) to 11 (when she worked as a higher executive with post-college education), with a mean of 44 (SD = 28). For those subjects for whom information about father’s education and profession was available, the correlation coefficient between paternal and maternal SES was calculated, revealing a strong positive two-tailed correlation (r = 0.783, p<0.001). For the purpose of brain-SES regression analyses, the scale of SES values was inverted, so that higher scores indicate higher SES.

**Table 1:** Hollingshead occupation and education codes.

Code	Occupation	Education
1	Higher executive, major professional, etc.	Post college
2	Business manager, etc.	College graduate
3	Administrative personnel, etc.	Part college or post high school training
4	Clerical and sales, technician, etc.	High school graduate
5	Skilled manual	Part high school
6	Machine operators, semi-skilled	Grammar school graduate
7	Unskilled	Part grammar school
8	Never employed	

### Cognitive Tests

A test battery was administered to all children in order to assess their performance in various cognitive tasks focusing on reading abilities. Reading ability was evaluated through two tests: Alouette [23], a standardized text reading fluency test in French for children aged between 6 and 16 years, and LUM (Lecture en une minute [24]), a one-minute word reading test. Phonological skills were assessed by a phoneme and syllable deletion task in pseudo-words (EVALEC [25]), and by a rapid automatized naming task (RAN) for pictures. Verbal skills were assessed by a test of picture naming (DEN48 [26]) and the verbal comprehension index from the WISC-IV battery [27]. Working memory was assessed by the digit span subtest of WISC-IV and by a word span task, where subjects were asked to repeat a series of digits or words in a sentence. Finally, visuo-spatial processing skills were tested by administering the Block Design subtest of WISC-IV and a test of visual search (the bells test [28]) in which the child has a limited time to identify as many target objects as possible among distracting items.

The scores in these tests were converted into z-scores relative to the sample mean and standard deviation, and 5 composite measures were constructed by averaging the relevant z-scores: literacy (text reading fluency and one-minute word reading), phonology (phoneme and syllable deletion and rapid automatized naming), verbal skills (picture naming and verbal comprehension), working memory (Digit span and word span) and visuo-spatial processing (Block Design and visual search).

### Imaging Procedure

All participants were familiarized with the MRI equipment in a mock scanner prior to the actual neuroimaging session. Whole brain images were then acquired on a 3-T Siemens Trio Tims MRI scanner using a 12-channel head coil. T1-weighted images were acquired with the following specifications: acquisition matrix: 256×256×176, TR = 2300 ms, TE = 4.18 ms, flip angle = 9 deg, field of view = 256 mm, voxel size: 1×1×1 mm. Diffusion images were acquired using an echo planar imaging sequence with 30 diffusion sensitized gradient directions (b-value = 1000 s/mm^2^; TR = 9500 ms; TE = 86 ms; square field of view = 240 mm; acquisition matrix: 128×128; voxel size: 1.9×1.9×3 mm; 40 slices with no inter-slice gap). During these sequences children watched cartoons.

### Analysis of Gray Matter

There are two well-established techniques to analyse gray matter correlates of subject characteristics. VBM is a widely used automated technique to detect voxel-by-voxel changes of gray matter volume, which involves registration of individual brain images to a template brain. SBM is an alternative method for probing gray matter changes, which maintains each subject in the native space and uses alignment based on cortical folding patterns. Additionally, it allows the respective contributions of cortical thickness and surface area to be determined. The two methods allow for the analysis of T1 images and use similar segmentation approaches, but they differ both in registration and in the way analyses are performed (in our case, voxel-based volumetric versus regional surface-based measurements). These differences could account for potential inconsistencies in outcome: therefore, we chose to employ both methods and report convergent findings in order to maximise the robustness of our results.

### VBM Analysis

For data preprocessing and analyses we used SPM8 software (Wellcome Trust Centre for Neuroimaging, London, UK, http://www.fil.ion.ucl.ac.uk/spm) run in MATLAB 7.1 (Mathworks, Sherborn, MA, USA). T1-weighted scans were segmented automatically into different tissue classes – gray matter (GM), white matter (WM) and non-brain (CSF, skull), using the ‘New Segmentation’ option in SPM8 [29]. In the segmentation step the tissue probability maps were taken from a customized pediatric brain template specific to the group characteristics (e.g. age and gender) generated using Template-O-Matic toolbox (http://dbm.neuro.uni-jena.de/software/tom/). The Diffeomorphic Anatomical Registration Through Exponentiated Lie Algebra (*DARTEL*) algorithm was then used to create a study-specific template. DARTEL works by aligning gray matter among the images, while simultaneously aligning white matter. This is achieved by generating its own increasingly crisp average template data, to which the data are iteratively aligned. This procedure begins by creating a mean of all the images, which is used as an initial template. Deformations from this template to each of the individual images are computed, and the template is then re-generated by applying the inverses of the deformations to the images and averaging [30]. This step was followed by affine registration of the GM maps to the Montreal Neurological Institute space scaling the GM probability values with the Jacobian determinants to ensure that the total signal in each tissue class remained constant (i.e. ‘modulation’) [31]. Spatial normalisation expands and contracts some brain regions and the information about local shearing, stretching, and rotation of voxels when an image is spatially normalized to a template is encode in the Jacobian matrices. Modulation involves scaling by the amount of contraction, so that the total amount of grey matter in the modulated GM remains the same as it would be in the original images [31]. Finally the data was smoothed with 4-mm full-width at half maximum Gaussian kernel.

A whole brain multiple regression analysis for all 23 subjects was performed with SES as regressor of interest and age, gender and total intracranial volume (TIV) as nuisance variables. TIV was calculated for each individual by summing up voxel values of the gray matter, white matter and CSG segmented in native space. Statistical significance thresholds were applied at the voxel-level (p<0.001, uncorrected). Results for the whole brain analysis were obtained using non-stationary correction (p<0.05 cluster extent threshold), which is crucial to adjust cluster sizes according to local roughness [32].

### SBM Analysis

Cortical surface-based reconstruction and volume segmentation were performed using the Freesurfer image analysis suite (version 4.5.0, http://surfer.nmr.mgh.harvard.edu/), which has been described and validated in previous papers [33], [34]. In brief, non-brain tissue is removed using a hybrid watershed/surface deformation procedure, after which the segmentation of the subcortical white matter and deep gray matter structures is performed. After intensity normalisation, the gray/white matter boundary is identified and tessellated. This surface is further used by a surface deformation algorithm aimed at optimally placing the gray/white and gray/CSF borders with sub-millimeter precision. Thereby, topologically correct polygonal mesh models of the cortical surfaces are created. The cortical models for all subjects are registered to a common surface template through a high-resolution surface-based averaging technique based on cortical folding patterns [35]. Parcellations grounded on gyral and sulcal structure (74 per hemisphere) are mapped from the template to each subject’s native space using a high-dimensional spherical morphing procedure [36]. Finally, a number of surface-based measures are calculated for each parcellation unit, such as gray matter volume, cortical thickness, cortical surface area and gyrification [37].

Failures of the automated processing stream can be observed, due to movements artifacts and signal intensity variation, leading to the introduction of segmentation errors (for instance, skull stripping failure or inclusion of dura or blood vessels into the pial surface). In our sample, qualitative and quantitative evaluations of the quality of segmentation led us to discard the cortical reconstructions for 2 subjects out of 23. Therefore, 21 children were included in this analysis. For each subject, a measure of the volume of each subcortical structure was obtained, as well as individual values of gray matter volume, cortical thickness, and cortical surface area for each parcellation unit. All these data, together with an estimation of total intracranial volume (TIV), mean hemispheric thickness and hemispheric total surface area, were extracted for each subject and analyzed in SPSS (version 10, SPSS Inc., Chicago IL). Stepwise regression analyses were run with age, gender and the global measure (TIV, mean hemispheric thickness or mean hemispheric surface area for volume, thickness and surface area measurements, respectively) as independent variables in a first block, and with SES in a second block.

### Analysis of Local Gyrification Pattern

The potential influence of SES on the cortical folding of the brain was also explored, as another way of assessing brain structural variability. It has been shown that cortical gyrification is affected in certain developmental and psychiatric disorders [38], [39] and that cortical complexity increases in frontal regions during development in healthy subjects [40].

A mean gyrification index [37] over each parcellated region of the Destrieux atlas was calculated, by computing the area ratio between an outer hull, tightly warping the brain, and the cerebral surface, thus giving a measure of surface area buried into sulci. Greater gyrification index could indicate deeper sulci and/or more gyrified/complex cortical patterns.

### Analysis of White Matter

Diffusion image preprocessing was performed using the BrainVISA software (http://brainvisa.info). First, images were motion-corrected using two successive steps: 1) automated detection of slices affected by intra-slice motion, and correction using spherical-harmonics decomposition of the diffusion signal; 2) standard correction for eddy current distortions and realignment of diffusion-weighted volumes, misregistered due to inter-volume motion. Second, whole-brain maps of fractional anisotropy (FA), mean diffusivity (MD), radial and axial diffusivities were created by fitting a tensor model to the diffusion volumes.

For the analysis of the whole-brain white matter tract integrity, the tract based spatial statistics (TBSS) standard protocol was applied as implemented in FSL [41]. The mean FA image was created and thinned to obtain a mean FA skeleton, which represents the centre of all tracts common to the group. This mean skeleton was then thresholded at FA>0.25 to reduce the likelihood of partial volume effect at the borders between tissue classes or cross-subject image misalignment. Next each subject’s aligned FA data was projected onto this skeleton and the resulting data was entered into voxelwise cross-subject statistics. The nonlinear warps and skeleton projection were then also applied to other DTI maps (mean, radial and axial diffusivities).

For voxel-wise regression analyses, a nonparametric permutation test with 5000 random permutations was performed with SES as regressor of interest and age and gender as nuisance variables. Threshold-Free Cluster Enhancement (TFCE) was used to obtain skeletal voxels significantly related to SES at p<0.05, after correcting for multiple comparisons across space using permutation testing.

## Results

### SES Correlations with Demographic and Cognitive Variables

**Figure 1:**
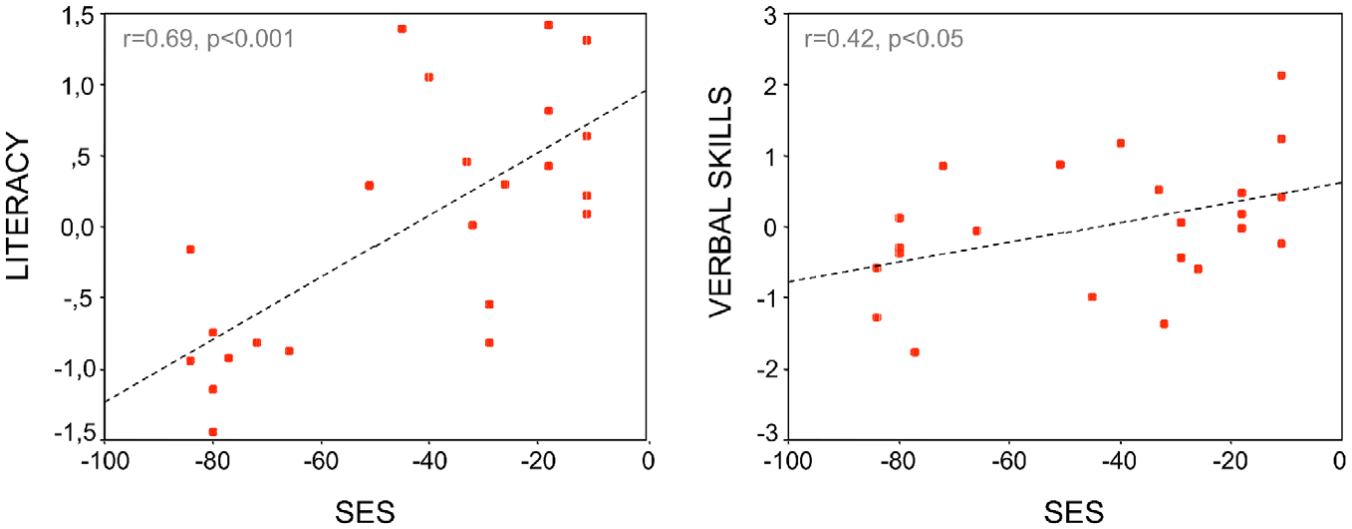
Correlations between SES and literacy and verbal skills.

We found significant correlations between SES and composite scores of literacy (r = 0.699, P<0.001) as well as verbal skills (r = 0.422, P = 0.045, Figure 1), consistently with previous findings [5], [8], [42]. Correlations with memory (r = 0.362, P = 0.09) and visuo-spatial processing (r = 0.358, P = 0.094) composite scores did not reach significance. There was no correlation between SES and phonological skills (P>0.25). Importantly, the correlation between SES and the non-verbal IQ subtest (Block Design) was not significant (P>0.4), suggesting that SES scores are not mere reflection of children’s IQ. We then tested whether SES was related to demographic variables and found no correlation between SES and age or birth weight. SES did not differ between sexes either.

### Association between SES and Gray Matter Properties

To examine the association between SES and gray matter volume we used two methods – voxel (VBM) and surface-based morphometry (SBM) which differ in terms of underlying assumptions, image processing and outcome measures, and were therefore used to cross-validate our results. No correlation was found between SES and global brain measures, i.e. total intracranial volume (TIV), mean hemispheric thickness and total surface area. However, local gray matter volume differences were found to be related to SES with both approaches.

**Figure 2:**
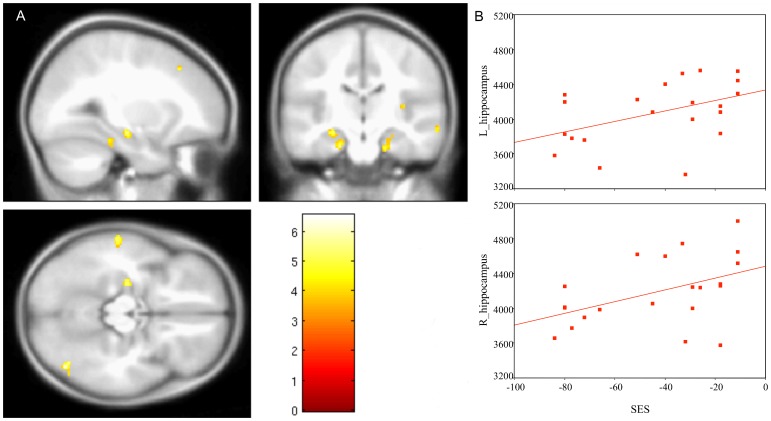
Gray matter volumes correlated with SES. A) VBM results displayed on a customized pediatric brain template; the color scale represents T-values; B) left and right hippocampus volume changes as a function of SES (from SBM).

VBM analysis revealed significant positive correlations between SES and local gray matter volumes in bilateral clusters including hippocampi and parahippocampal gyri, middle temporal gyri, insula, as well as in the left fusiform gyrus, right inferior occipito-temporal region and left superior/middle frontal gyrus (see Figure 2A and Table 2 for details). No region was negatively correlated with SES.

**Table 2:** Brain regions related to SES revealed by VBM.

REGION	MNI coordinates			Z score	Cluster size(voxels)
	x	y	z		
L hippocampus/parahipp. gyrus	−20	16	−26	4.18	127
L hippocampus	−26	−16	−12	4.16	72
L middle temporal gyrus	−66	−24	−14	4.01	90
L fusiform gyrus	−33	−27	−35	3.95	123
R inf. occipito-temporal	45	−70	−12	4.61	123
R middle temporal gyrus	64	−15	−9	4.34	64
R hippocampus/parahipp. gyrus	20	−19	−27	4.13	295
R hippocampus (incl. in the cluster)	26	−10	−17	3.76	44
L sup./mid frontal gyrus	−18	29	42	4.08	70
R insula	39	−10	10	4.01	132
L insula	−39	−9	7	4.34	125

Only clusters surviving P<0.05 non-stationary cluster correction for extent and greater than 60 voxels are reported. The regions identified by both procedures (VBM and SBM) are underlined.

SBM analysis confirmed some of these findings: this was the case for the volumes of bilateral hippocampi, bilateral middle temporal gyri, left fusiform gyrus (medial occipital-temporal and lingual sulcus in Destrieux et al.’s atlas) and right inferior occipito-temporal region (right inferior temporal sulcus). In these regions, a partial overlap between VBM clusters and SBM areas was observed (see Table 3). In addition, analyses based on SBM revealed a number of regions for which thickness and/or surface area correlated with SES, but with no counterpart in the VBM analysis.

**Table 3:** Brain regions related to SES revealed by SBM.

REGION	Volume	Thickness	Surface
L hippocampus	t = 2.23, P = 0.039, R^2^ = 0.43		
L middle temporal gyrus (38)	t = 2.11, P = 0.05, R^2^ = 0.63		
L medial occipital-temp	t = 2.49, P = 0.023, R^2^ = 0.39		
and lingual sulcus (61)			
R inf temporal sulcus (72)	t = 2.74, P = 0.013, R^2^ = 0.64		t = 3.93, P<0.001, R^2^ = 0.77
R middle temporal gyrus (38)	t = 3.74, P = 0.002, R^2^ = 0.79		
R hippocampus	t = 2.22, P = 0.04, R^2^ = 0.40		
L fronto-marginal gyrus and sulcus (1)		t = −4.38, P<0.001, R^2^ = 0.57	
L mid frontal sulcus (53)		t = −2.89, P = 0.009, R^2^ = 0.27	
L angular gyrus (25)	t = 2.94, P = 0.009, R2 = 0.56	t = 2.23, P = 0.039, R^2^ = 0.34	
L gyrus of Heschl (33)		t = 2.89, P = 0.01, R^2^ = 0.46	
L post-ventral cingulate gyrus (10)		t = 2.77, P = 0.012, R^2^ = 0.25	
L intraparietal sulcus (56)		t = −2.19, P = 0.042, R^2^ = 0.65	
L anterior occipital sulcus (59)		t = 2.12, P = 0.048, R^2^ = 0.29	
L superior occipital gyrus (20)			t = −2.41, P = 0.027, R^2^ = 0.67
R inferior frontal sulcus (52)	t = 2.21, P = 0.041, R2 = 0.53		t = 2.29, P = 0.034, R^2^ = 0.38

R^2^ stands for adjusted R-square in the multiple regression analysis. Numbers in parentheses refer to cortical regions in Destrieux’s atlas. The regions identified by both procedures (VBM and SBM) are underlined.

**Figure 3:**
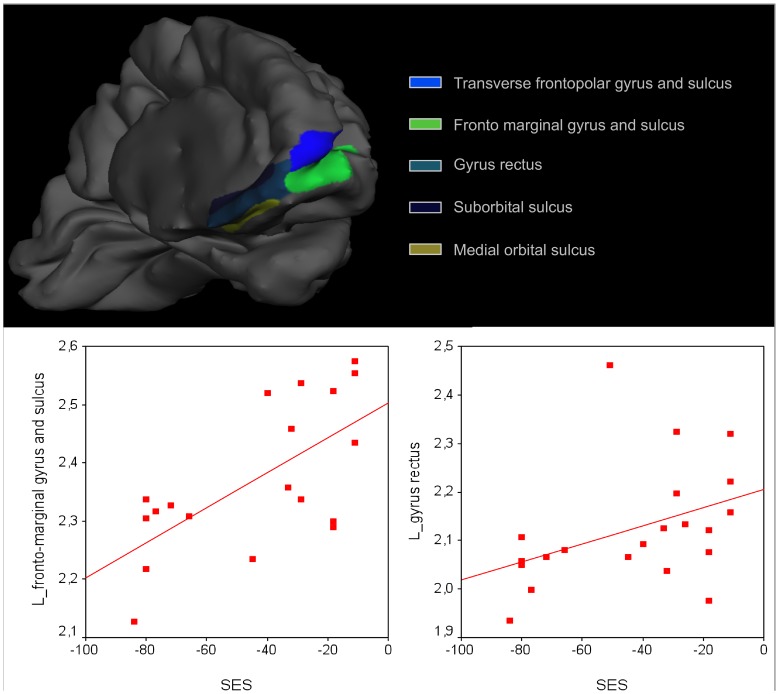
Left hemispheric anterior frontal regions showing a positive correlation between gyrification index and SES.

We found positive correlations between gyrification and SES in the left hemisphere (see Figure 3), specifically in anterior frontal regions: fronto marginal gyrus and sulcus (t = 3.85, P<0.001), gyrus rectus (t = 3.56, P = 0.003), suborbital sulcus (t = 3.38, P = 0.004), transverse frontopolar gyrus and sulcus (t = 3.15, P = 0.006), medial orbital sulcus (t = 3.09, P = 0.007).

### Association between SES and White Matter Microstructure

To evaluate the effect of SES on the whole-brain white matter architecture we used tract based spatial statistics (TBSS) on DTI images. This revealed no significant correlation between white matter properties and SES, neither for fractional anisotropy nor for any other diffusion index (mean, radial and axial diffusivity). This remained unchanged even when the threshold was relaxed to P<0.005 uncorrected.

## Discussion

Parental socioeconomic status has been shown to affect cognitive functions in children. Here, we obtained further confirmation of these effects, and we provide evidence that SES is also associated with children’s brain structure. These results, obtained in healthy children in a developed country, suggest that the brain structure can be associated with unfavorable environmental conditions, even when they do not reach extreme deprivation and stress.

At the cognitive level, in line with previous studies [6], [7], [8], we found positive correlations between SES, reading and verbal abilities, confirming that language is one of the cognitive domains most affected by SES. Correlations with other cognitive skills were not significant, although there were trends concerning working memory and visuo-spatial skills that might require greater statistical power. We found no correlation between SES and phonological skills in our sample of children, suggesting that SES does not influence all language related skills uniformly. Most previous studies have shown that that SES and PA are associated [7], [43], [44], however negative findings have also been reported [6], [45]. Given that children with reading difficulties were excluded from our sample, it is possible that the range of phonological skills may have been too narrow to reveal a significant effect.

At the brain level, positive correlations were observed between SES and local gray matter volumes in bilateral hippocampi, middle temporal gyri, left fusiform and right inferior occipito-temporal gyri. These relations held consistently across two different approaches to the analysis of structural MRI data, namely voxel- and surface-based morphometry. Overall they confirmed at least a subset of our predictions concerning brain regions associated with cognitive functions known to be affected by SES.

We found consistent correlations between SES and bilateral hippocampi, extending to parahippocampal gyri, in agreement with the well-documented association of these structures with memory performance as well as with prenatal and early stress. Despite using a whole brain approach with suitable corrections for multiple tests, we were able to confirm the relation between hippocampal volume and SES previously reported in a ROI-based study [13]. Although a prediction was made of an influence of SES on left perisylvian regions associated with language [3], no such association was revealed in a convergent manner by the two methods. On the other hand, we found a consistent relation between SES and the left temporal-occipital areas (fusiform and middle temporal gyrus) that are more specifically associated with written language [46]. This is also a highly plausible relationship, given the strong effect of SES on reading ability [6], [42], which we replicated in the present sample. Additionally, in the left medial prefrontal cortex, a positive correlation was found between SES and gyrification. It has been shown that the fractal complexity of gyral/sulcal convolutions increases bilaterally in prefrontal areas between 6 and 16 years of age [40]. Future studies on larger samples, comparing different cortical complexity indices, will be necessary to uncover whether slower developmental trajectories more generally characterize low SES children, as has been suggested functionally on the basis of EEG resting state activity [47], [48].

Some of the brain areas that we found to be related to SES did not correspond to any a priori prediction, and remain to be both confirmed and fully interpreted. These include the right inferior occipito-temporal region and the right middle temporal gyrus. The former could be associated with literacy as previous studies revealed its functional involvement in reading acquisition [49]. It is worth noting that all the correlations that we found between SES and gray matter dimensions using the two methods were positive. This would certainly not be expected by chance, and suggests that a favourable environment is generally associated with larger quantities of gray matter.

Finally, when we examined the relationship between SES and white matter microstructure as estimated from diffusion sequences, we found no significant correlation in any part of the brain, in line with a previous study on adults [15]. This completely null result stands in remarkable contrast with the abundance of positive correlations found with gray matter in various regions, and certainly suggests that if SES has any effect on white matter architecture, it must be much smaller than its effects on gray matter.

An obvious limitation of the present study is its small sample size. Nevertheless, the fact that we were able to find significant and matching effects using two different whole-brain approaches (VBM and SBM) and that at least some of these effects met a priori predictions, suggests that we had adequate statistical power to detect at least the most reliable effects. This may be in part attributed to the relatively narrow age range, which may have limited variations due to brain development, and to the large SES range obtained through the selection of the sample. Additional studies on larger samples of children will be needed to confirm the brain-SES correlations found here. Such work should employ a wider range of cognitive measures to fully characterize the effects of SES and disentangle the potential cognitive mediators of brain-SES links. Furthermore, additional measurements of income, stress, parental care or nutrition could shed more light on the mechanisms by which SES influences the brain. Indeed SES is a rather fuzzy construct which is easily accessible but that captures many distinct sources of variation of very different nature. Further work should certainly aim to break it down into more elementary components.

Another caveat is that the present study does not allow us to establish the direction of causality between SES, brain morphology and cognitive functions. In addition, we have no direct proof that the link between parental SES and children’s brain is entirely environmentally mediated. Indeed it is expected that part of the variance evidenced here may be genetically mediated. However, there is considerable evidence for direct environmental effects in other species, and some of the effects found here (e.g. the hippocampus) are precisely those that would be expected from environmental influences. In rodents the quality of post-natal environment i.e. maternal care, cognitive stimulation (environmental enrichment), can affect neural and cognitive functioning [18]. Increased maternal care results in better spatial learning/memory skills and heightened synaptogenesis in the hippocampus. This relationship seems to be a direct one as it has also been evidenced in a cross-fostering study [50]. Environmental enrichment has also been shown to increase neurogenesis in the hippocampus and even reverse the negative effects of reduced maternal care on hippocampal-dependent spatial and non-spatial learning [51]. Thus it seems that hippocampus might be one of the most sensitive brain structures to variations in post-natal environment such as heightened amounts of stress and reductions in environmental stimulation in low SES families.

The results of the present study should therefore be taken as preliminary findings and can be considered as guidance for future work on brain regions associated with socioeconomic status. A recent review [4] warned that looking for MRI correlates of SES would not necessarily add much to our understanding of the effects of SES, beyond what we already know from SES-behavior correlations. While this view may be tenable in the case of fMRI activations that are mere neurofunctional correlates of cognitive performance, we find that structural MRI does go further. Here, we have shown that, beyond functional changes, socioeconomic status is actually related with widespread modifications on children’s brain structure. These modifications are largely consistent with predictions drawn from functional considerations, nevertheless structural MRI is opening a more direct window onto the potential biological mechanisms underlying the effects of SES on cognition.

Finally, it should be emphasized that our findings do not by themselves carry any direct social implication. It is already well-known that SES has effects on cognitive development. Understanding the specific brain mechanisms through which these effects may occur is a fascinating prospect, however this does not change courses of action that may be taken. Irrespective of neural mediators, reducing the detrimental cognitive effects of some factors associated with low SES implies identifying those factors and trying to mitigate them. Thus, policies can be adopted to reduce cases of fetal and child malnutrition, to generally improve fetal and infant health, and to compensate the effects of socio-cultural disadvantages through early schooling and specific educational programs [52], [53].
