# Correction: The Influence of Socioeconomic Status on Children’s Brain Structure

**DOI:** 10.1371/annotation/47661de2-2c53-4396-9f88-06b5ad233566

**Published:** 2012-10-18

**Authors:** Katarzyna Jednoróg, Irene Altarelli, Karla Monzalvo, Joel Fluss, Jessica Dubois, Catherine Billard, Ghislaine Dehaene-Lambertz, Franck Ramus

There was an error in affiliation 1 for authors Katarzyna Jednoróg, Irene Altarelli, and Franck Ramus. Affiliation 1 should be: Laboratoire de Sciences Cognitives et Psycholinguistique, Département d’Etudes Cognitives, Ecole Normale Supérieure, École des hautes études en sciences sociales, Centre national de la recherche scientifique, Paris, France 

